# When is an obscurin variant pathogenic? The impact of Arg4344Gln and Arg4444Trp variants on protein–protein interactions and protein stability

**DOI:** 10.1093/hmg/ddab010

**Published:** 2021-01-12

**Authors:** Atsushi Fukuzawa, Daniel Koch, Sarah Grover, Martin Rees, Mathias Gautel

**Affiliations:** Randall Centre for Cell & Molecular Biophysics, King's College London, 18–20 Newcomen Street, SE1 1UL, UK; Randall Centre for Cell & Molecular Biophysics, King's College London, 18–20 Newcomen Street, SE1 1UL, UK; Randall Centre for Cell & Molecular Biophysics, King's College London, 18–20 Newcomen Street, SE1 1UL, UK; Randall Centre for Cell & Molecular Biophysics, King's College London, 18–20 Newcomen Street, SE1 1UL, UK; Randall Centre for Cell & Molecular Biophysics, King's College London, 18–20 Newcomen Street, SE1 1UL, UK

## Abstract

Obscurin is a giant muscle protein that connects the sarcomere with the sarcoplasmic reticulum, and has poorly understood structural and signalling functions. Increasingly, obscurin variants are implicated in the pathophysiology of cardiovascular diseases. The Arg4344Gln variant (R4344Q) in obscurin domain Ig58, initially discovered in a patient with hypertrophic cardiomyopathy, has been reported to reduce binding to titin domains Z8-Z9, impairing obscurin’s Z-disc localization. An R4344Q knock-in mouse developed a cardiomyopathy-like phenotype with abnormal Ca^2+^-handling and arrhythmias, which were attributed to an enhanced affinity of a putative interaction between obscurin Ig58 and phospholamban (PLN) due to the R4344Q variant. However, the R4344Q variant is found in 15% of African Americans, arguing against its pathogenicity. To resolve this apparent paradox, we quantified the influence of the R4344Q variant (alongside another potentially pathogenic variant: Arg4444Trp (R4444W)) on binding to titin Z8-Z9, novex-3 and PLN using pull-down assays and microscale thermophoresis and characterized the influence on domain stability using differential scanning fluorimetry. We found no changes in titin binding and thermostability for both variants and modestly increased affinities of PLN for R4344Q and R4444W. While we could not confirm the novex-3/obscurin interaction, the PLN/obscurin interaction relies on the transmembrane region of PLN and is not reproducible in mammalian cells, suggesting it is an *in vitro* artefact. Without clear clinical evidence for disease involvement, we advise against classifying these obscurin variants as pathogenic.

## Introduction

**Figure 1:**
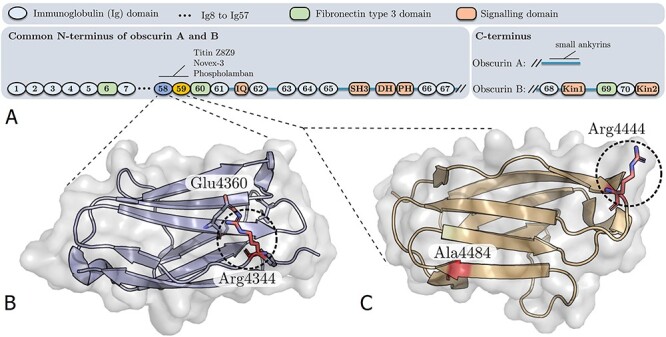
(**A**) Domain composition of large obscurin isoforms A and B. Position of residues affected by mutation are shown in the Ig58 (**B**) (PDB-ID: 4RSV) and Ig59 (**C**) (PDB-ID: 5TZM) structures of obscurin.

Obscurin is a giant muscle protein that connects the contractile unit of muscle cells, the sarcomere, with their intracellular calcium storage, the sarcoplasmic reticulum (SR) (1,2). Like many other large sarcomeric proteins, it exists as different splicing isoforms (3,4). The two largest isoforms, obscurin A and obscurin B, are mainly composed of immunoglobulin-like (Ig) domains, but feature several signalling domains in their C-terminal third: a calmodulin binding IQ-motif, an SH3 domain and a DH/PH domain tandem typical for Dbl-family Rho-GEFs (Fig. 1A). Obscurin B additionally features two kinase domains. Obscurin A, in contrast, has putatively unstructured regions at the C-terminus that can bind to small ankyrins in the SR membrane, thereby connecting the M-band of the sarcomere with the SR (2,3,5,6). At least two smaller isoforms exist, one of which features the SH3-DH/PH triplet, the other of which contains the kinase domains (3,4). The multitude of domains and interactions (e.g. with titin Z8-Z9 and small ankyrins) (1,5,6) suggests that obscurin has both structural and signalling functions, which are only beginning to emerge. The picture is further complicated by developmentally specific localization patterns and a multitude of post-translational modifications (1,7,8).

Despite our limited understanding of the protein, genetic obscurin variants are increasingly implicated into the pathophysiology of muscular and cardiovascular diseases (9–13). The first variant suggested to be involved in the pathophysiology of cardiomyopathy is the R4344Q mutant in obscurin Ig58. Discovered in a patient with hypertrophic cardiomyopathy by Arimura *et al*., their data suggested that the R4344Q mutation impairs binding to Titin Z8-Z9 and reduces incorporation of transfected obscurin Ig58-Ig59 into the sarcomeric Z-disc of neonatal rat cardiomyocytes (9). More recently, a homozygous R4344Q knock-in mouse created by the Kontrogianni–Konstantopulous group developed cardiac arrhythmias under sedentary conditions and a DCM-like phenotype upon pressure overload, which the authors attributed to an increase in the affinity, as judged by co-immunoprecipitation, for a newly identified interaction between obscurin and phospholamban, a small inhibitory peptide of the SERCA pump in the SR membrane (14). A different obscurin mutation in the neighbouring Ig59 domain, R4444W, has also been described to impair the binding to Titin Z8-Z9 and is suggested to increase the penetrance of distal muscular dystrophy when co-inherited with a Filamin-C frameshift mutation (13). Of note, this co-inheritance of two alleles on two different chromosomes would have had to occur over four generations to explain the phenotype in the described family (13).

Crystal structures of obscurin Ig58 and Ig59 are available from the protein data bank. R4344 in Ig58 is surface exposed and forms a salt bridge with Glu4360 (Fig. 1B). Mutation of arginine to glutamine would result in a loss of the salt bridge, but would not be predicted to affect the folding of the domain. R4444 in Ig59 is also surface exposed, and the side chain does not mediate polar contacts with other residues (Fig. 1C). Mutation to the large, hydrophobic residue tryptophan is not predicted to sterically hinder nearby residues or affect domain folding, but tryptophan is more typically found in the core of proteins.

Curiously, and at odds with the findings of Arimura *et al.* and Hu *et al.*, the R4344Q variant is found in up to 15% of African Americans and has a high overall mean allele frequency of 0.01165, which prompted several researchers to question the pathogenicity of this variant (10,15). To resolve the apparent paradox between the previous *in vitro* findings and the population genetics, we characterized the effect of the R4344Q variant on protein–protein interactions and protein domain stability, using quantitative biochemical and biophysical approaches. We also included the R4444W variant in our study as a comparison, since it was reported to affect some of the same interactions as R4344Q and to be potentially pathogenic. While our data contradict the findings by Arimura *et al.* and Hu *et al.*, it supports the argument that the R4344Q variant is too common to be pathogenic. Our findings should thus help to refine diagnostic fidelity in patients carrying obscurin variants.

## Results

### Effect of the R4344Q variant on protein–protein interactions

**Figure 2:**
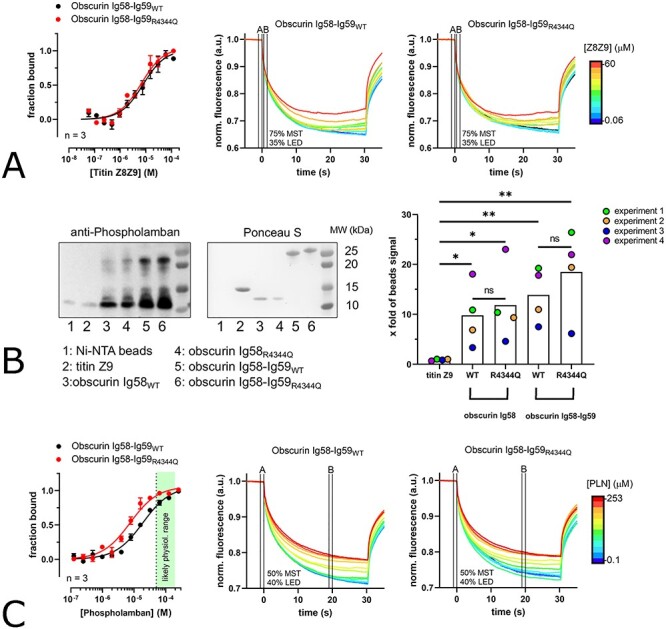
Effect of the obscurin R4344Q variant on protein–protein interactions. (**A**) MST shows no significant difference between wild-type and R4344Q variant for titin Z8-Z9 binding. Binding curves based on signal at Lane B (MST-laser on) divided by signal at Lane A (MST-laser off) in the MST traces. (**B,C**) Pulldown and MST data showing binding of human full-length phospholamban to obscurin Ig58 and Ig58-Ig59. MST data show statistically significant but moderate affinity increase for the R4344Q variant.

To study and quantify any potential effect of the R4344Q variant on protein–protein interactions, we used microscale thermophoresis (MST), which measures how a binding partner influences the migration of a labelled protein along a temperature gradient (17). We found no significant difference in the affinity for titin Z8-Z9 between wild-type obscurin Ig58-Ig59 (*K_d_* = 7.8 ± 1.7 μM) and Ig58-Ig59_R4344Q_ (*K_d_* = 6.6 ± 1.2 μM) (Fig. 2A). We also used isothermal titration calorimetry to verify the affinities based on reaction heat and found similar *K_d_* values (data not shown).

Since obscurin Ig58-Ig59 has been reported to interact with novex-3, a small titin isoform, we also sought to characterize whether the R4344Q variant might influence the affinity towards novex-3 (18). However, we were not able to detect an interaction between obscurin Ig58-Ig59 and the obscurin binding region of novex-3 (ObB, residues 4991 to 5187) by MST (Supplementary Material, Fig. S1) or pull-down assays using novex-3 ObB expressed in HEK293A cells (Supplementary Material, Fig. S2). Bang *et al*. identified this interaction in the yeast two hybrid system and then confirmed it in an *in vitro-transcription/translation* pulldown assay, using a cell-free eukaryotic expression system based on reticulocyte lysate for producing novex-3 ObB. Although the ObB sequence used by Bang and colleagues shows only minor differences to our sequence (Supplementary Material, Fig. S3), we cannot rule out that these differences, the different cell types used for producing the ObB (reticulocytes vs. *E. coli* and HEK293A) or other factors play a role for this interaction.

Next, we sought to characterize the binding between obscurin Ig58 or Ig58-Ig59 and phospholamban. First, we tried to replicate the findings by Hu *et al.* by performing similar pull-down assays with recombinant/synthetic proteins. As shown in Figure 2B, we could confirm an interaction between obscurin Ig58 or Ig58-Ig59 and phospholamban *in vitro*. In contrast to Hu *et al.,* however, we found no significant increase in binding for the R4344Q variant. We also found that Ig58-Ig59 interacts both with monomeric (low MW bands) and pentameric phospholamban (higher MW bands). Quantifying the binding affinities using MST, we found a statistically significant but modest difference amounting to a 2.5-fold decreased *K_d_* for the R4344Q variant (Fig. 2C; *K_d_* wild-type = 18.9 ± 2 μM, *K_d_* R4344Q = 7.5 ± 1 μM). In agreement with our pull-down data, the binding curve is well described by a single binding-site model, indicating that all phospholamban protomers (i.e. also those in a pentamer) are available for binding obscurin.

### Effect of the R4444W variant on protein–protein interactions

**Figure 3:**
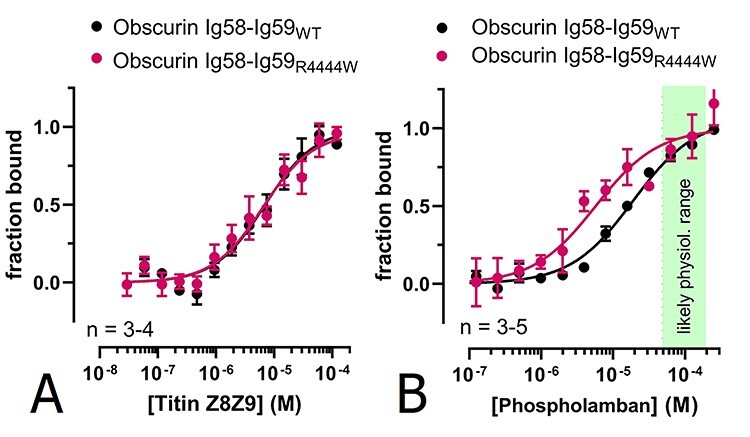
Effect of the obscurin R4444W variant on protein–protein interactions. (**A**) No effect can be observed on the interaction with titin Z8-Z9 as measured by MST. (**B**) MST analysis of the interaction between obscurin Ig58-Ig59_R4444W_ and phospholamban reveals an increase in affinity.

The R4444W variant of the obscurin Ig59 domain is reported to weaken the interaction between obscurin Ig58-Ig59 and titin Z8-Z9 (13). We therefore tested the influence of the R4444W variant on the interaction with titin Z8-Z9 and phospholamban. As shown in Figure 3A, we detected no difference in titin Z8-Z9 binding compared to the wild-type protein and Ig58-Ig59_R4344Q_ (*K_d_* = 6.4 ± 1.6 μM). Surprisingly, however, we found that the R4444W variant, too, has a higher affinity for phospholamban (*K_d_* = 5.7 ± 1.2 μM) than wild-type obscurin (Fig. 3B).

**Figure 4:**
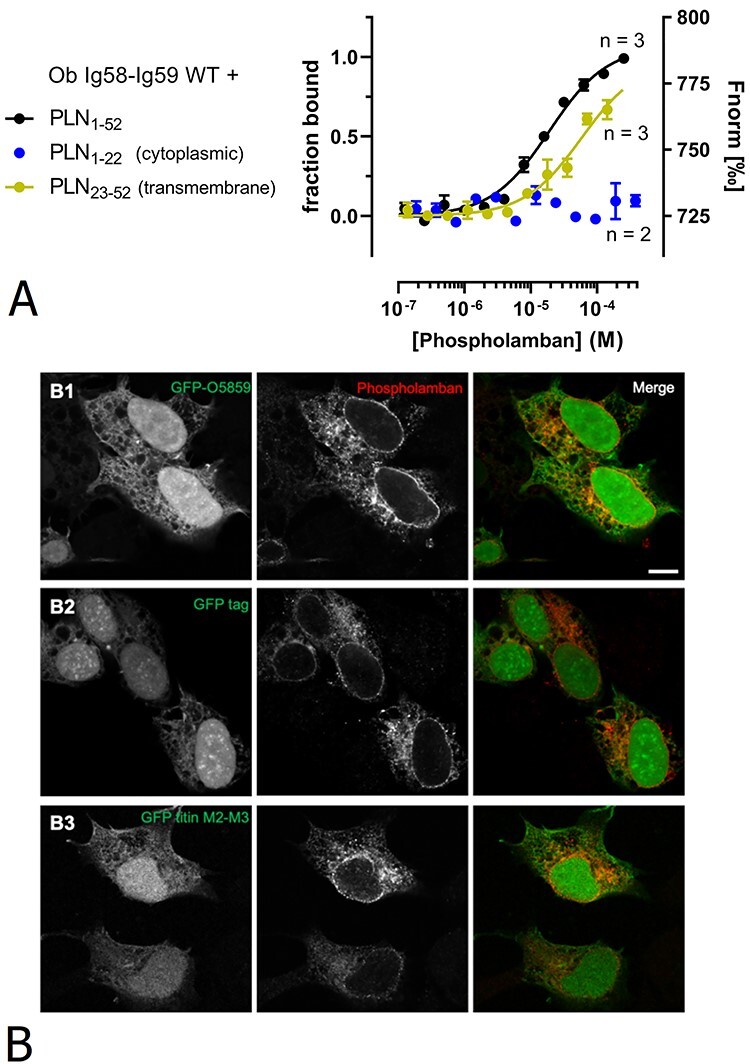
Characterization of the interaction between obscurin and phospholamban. (**A**) MST analysis using full-length phospholamban, cytoplasmic and transmembrane region. Data for the cytoplasmic region are displayed as normalized fluorescence (Fnorm) due to absence of binding. (**B**) Obscurin Ig58-59 did not show significant co-localization with phospholamban. HEK293A cells were co-transfected with phospholamban and B1) GFP-obscurin Ig58-Ig59, B2) GFP-empty or B3) GFP-titin M2-M3. Scale bar: 10 μm.

Based on structural modelling and computational docking, Hu *et al.* proposed that the R4344Q replacement stabilizes putative electrostatic interactions between obscurin Ig58 and the cytoplasmic region of phospholamban (14). The replacement of a hydrophilic and charged arginine with a hydrophobic tryptophan in the R4444W variant, however, seems inconsistent with such an interaction mode. Therefore, we characterized this interaction in more detail and tested if obscurin Ig58-Ig59 binds phospholamban’s cytoplasmic or transmembrane region in isolation. Interestingly, we found that obscurin binds to the transmembrane region (residues 23–52), albeit with a ≈3-fold weaker affinity (*K_d_* = 58.5 ± 1.7 μM) compared to full-length phospholamban. In contrast, we detected no interaction with the cytoplasmic region (residues 1–22) (Fig. 4A). The transmembrane region of phospholamban might not be accessible for obscurin binding *in vivo* when inserted in membrane. If it is, however, we reasoned that phospholamban might be able to recruit obscurin to membrane structures. To test this hypothesis, we co-transfected HEK293A cells with phospholamban and GFP-tagged obscurin Ig58-Ig59. Confocal microscopy showed that phospholamban is exclusively targeted to membrane structures, most likely ER and/or ER-derived vesicles, whereas obscurin Ig58-Ig59 exhibits unspecific and diffuse localization in both cytoplasm and nuclei (Fig. 4B, panel B1) without colocalizing with phospholamban-positive vesicles. GFP alone and also GFP-tagged titin M2-M3, which we used as a control for two tandem Ig domains, showed very similar, diffuse localizations as obscurin Ig58-Ig59 (Fig. 4B, panels B2 and B3). These results suggest that while the transmembrane region of phospholamban is capable of obscurin binding *in vitro,* when phospholamban is integrated into membrane structures of living cells, this interaction is abolished most likely because the transmembrane region is not accessible to obscurin.

### Effect of the R4344Q and the R4444W variant on thermostability

**Figure 5:**
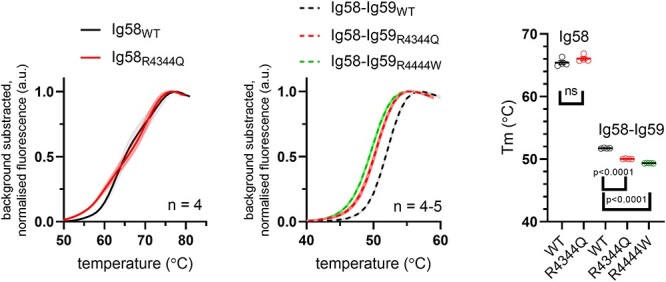
Effect of the obscurin R4344Q and R4444W variants on domain stability. Differential scanning fluorimetry for increasing temperatures shows no biologically relevant increase in protein domain unfolding for either variant when compared to the wild-type protein. Melting temperatures were determined by fitting melting curves to the Boltzmann equation.

The destabilization of protein domain folding can play a major causative role in the pathogenesis of cardiovascular and other diseases (19,20). Pathogenic destabilizing mutants in titin Ig-domains, for example, typically lead to differences in melting temperatures of >>10°C or result in insoluble protein expression (21,22). We therefore probed the influence of the R4344Q and R4444W variants on thermostability using differential scanning fluorimetry (DSF). Both the wild-type and variant domains were solubly expressed, and as shown in Figure 5, neither the R4344Q nor R4444W variant lead to a biologically relevant decrease of the protein’s melting temperature when compared to the wild-type protein. Thus, neither variant has a destabilizing effect on domain structure.

### Characterization of an R4344Q, A4484T double variant

**Figure 6:**
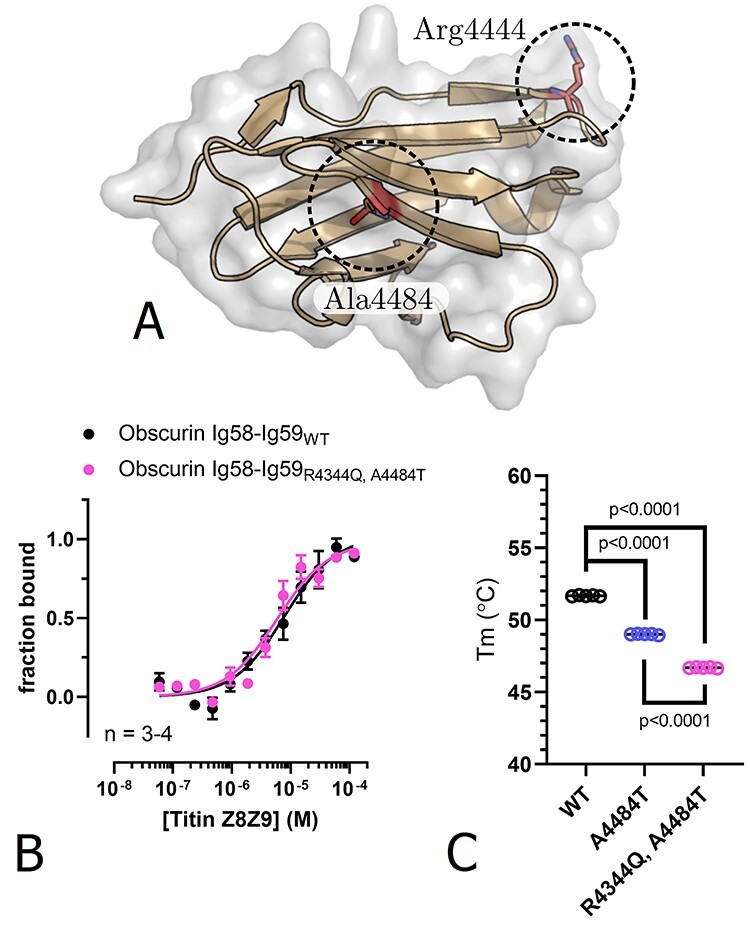
Characterization of the obscurin R4344Q, A4484T double variant. (**A**) Position of A4484 affected by mutation in the Ig59 structure of obscurin (PDB-ID: 5TZM). (**B**) No effect can be observed for the affinity to titin Z8-Z9 as measured by MST. (**C**) Thermostability of obscurin Ig58-Ig59WT, A4484T variant, and R4344Q, A4484T double variant.

Although the characterized variants are likely benign in isolation (see discussion), it is interesting to note that in the context of their discovery, both the R4344Q and the R4444W variant co-segregated with additional variants (9,13). This raises the possibility that the disease phenotype of the patients, in whom these variants were discovered, is the cumulative effect of multiple variants. For instance, the patient in whom the R4344Q variant was discovered was also a carrier of the A4484T variant on Ig59 in *cis* (Fig. 6A). Although Arimura *et al.* found no effect of the A4484T variant (9), we decided to test the influence of an R4344Q, A4484T double variant on the interaction with titin Z8-Z9 and on thermostability.

In agreement with Arimura *et al.*, we found that the double variant does not diminish the affinity for binding to titin Z8-Z9 (Fig. 6B). However, we found that the A4484T mutation alters the way how the titin Z8-Z9 ligand changes the MST signal: instead of increased normalized fluorescence upon ligand binding, as observed for obscurin Ig58-Ig59_WT_, R4344Q and R4444W, the double variant showed a decrease in fluorescence upon titin Z8-Z9 binding (Supplementary Material, Fig. S4A). Since the temperature jump signal that we used for characterizing this interaction is highly dependent on the chemical environment of the fluorophore (17), this could either indicate that the 4484 position lies in the vicinity of the labelling site, or that the A4484T substitution locally alters the conformation of the Ig58-Ig59 tandem without impairing the titin interaction. Moreover, we observed a moderate but consistent tendency of the double variant to form aggregates during the MST-assay (Supplementary Material, Fig. S4B). Next, we characterized the thermostability of the A4484T variant and the R4344Q, A4484T double variant. We found that the A4484T variant decreases the melting temperature by 2.7°C, resulting in a cumulatively 5°C lower melting temperature for the R4344Q, A4484T double variant compared to the wild-type protein (Fig. 6C). Although the observed *in vitro* differences are statistically significant, the magnitude of the effect remains far lower than observed in known disease-causing destabilizing mutations (21,22). Taken together, our data suggest that the R4344Q, A4484T double variant is unlikely to be pathogenic.

## Discussion

The loss or change of affinity of protein–protein interactions and the destabilization of protein domain folding belong to the major molecular mechanisms linking pathogenic genetic variants to disease phenotypes. In this study, we focused on obscurin domains Ig58-Ig59, which potentially bind to multiple proteins including titin Z8-Z9, phospholamban and novex-3 and for which pathogenic mutations were reported previously.

### The R4344Q variant

**Figure 7:**
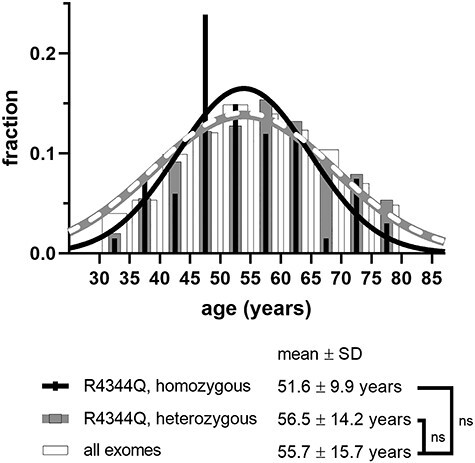
Age structure of homozygous and heterozygous carriers of the R4344Q variant. If the R4344Q variant would lead to significant cardiac disease (such as dilated cardiomyopathy), variant carriers could be expected to die earlier, resulting in a ‘left-shift’ of the age distribution of variant carriers. The absence of any significant change in the mean age compared to the overall population confirms that carriers of the R4344Q variant do not die earlier, indicating that the variant is not pathogenic.

Taken together, our data suggest that the only influence of the R4344Q variant is a 2.5-fold decreased *K_d_* for the interaction with phospholamban *in vitro*. Interestingly, our data further suggest that this interaction relies primarily on the transmembrane region of phospholamban and that it does not to occur in co-transfected mammalian cells. Our findings thus contest the physiological plausibility of this interaction and indicate that it is likely an *in vitro* artefact, as the transmembrane region will not be solvent accessible to a cytoplasmic Ig-domain. Consistent with an unspecific hydrophobic interaction, both obscurin variants substitute a hydrophilic arginine with a less hydrophilic residue. Even if one would assume that the interaction is physiological, the question arises whether a 2.5-fold decreased *K_d_* would be functionally relevant. At physiological phospholamban concentrations of >50 μM (23), the difference in affinity would result in at most 15% higher fractional saturation of obscurin binding sites with phospholamban (Fig. 2C, green shaded area). Since most obscurin binding sites would be already occupied by phospholamban at such concentrations, it seems implausible that a <15% increase would significantly impair whatever the function of this interaction would be. This is in excellent agreement with the genetic prevalence of this variant. Although Hu *et al*. proposed that this variant might contribute to the higher risk for cardiovascular disease among African Americans, prevailing socio-economic factors and other health risks among African Americans render a genetic ‘*obscurin ex machina’* an unnecessary explanation (14,24). Comparing the age structure of R4344Q carriers in the gnomAD database to the overall population shows that heterozygous carriers do not die earlier than the rest of the population and while the number of homozygous carriers in the database is too low to draw definite conclusions, the mere fact that it contains dozens of homozygous carriers, many of which live well beyond their sixth decade, argues against any significant pathogenicity (Fig. 7). Why then, on the other hand, do R4344Q knock-in mice develop cardiac arrhythmias and a DCM phenotype upon pressure overload (14)?

Let us assume, for the sake of the argument, that the interaction between phospholamban and obscurin would be a physiological one. An interesting clue to this puzzle then is the observation that there are several quantitative differences between murine and human calcium handling. Firstly, mice seem to have slightly lower phospholamban concentrations than larger species (25–27), thus the difference in fractional saturation of obscurin binding sites could be bigger for the R4344Q variant in mice. Secondly, the SERCA pump, the main regulatory target of phospholamban, is responsible for 70% of calcium removal in human cardiomyocytes, but for >90% of calcium removal in cardiomyocytes of small rodents such as mice (23). Combined with a 10-fold higher resting heart rate, any detrimental effects of the R4344Q variant could be much more pronounced in the murine heart. We thus speculate that the phenotype observed by Hu *et al.* might be specific to the physiology of the murine heart.

### The R4444W variant

The minor allele frequency of the R4444W variant is at 1.2 × 10^−4^ among the European/American population (13), more consistent with a potentially pathogenic mutation than the frequency of the R4344Q variant. In our hands, however, the R4444W variant did not exhibit any effect on protein–protein interactions either, contrary to the results of Rossi *et al*. who found a 15-fold increased *K_d_* for the interaction with titin Z8-Z9 due to the R4444W variant (13). Having studied this interaction using surface plasmon resonance (SPR), the authors explained this result with a strongly decreased *on*-rate constant based on their fit to the SPR data. This is surprising for two reasons: first, empirically, many single amino-acid substitutions typically have a far bigger impact on the *off*-rate rather than on the *on*-rate constant of simple binding reactions (28,29). This is intuitively plausible if one assumes binding to be limited by diffusion and orientation but dissociation to be limited by the strength of the molecular interactions between two molecules (30). Secondly, a decreased *on*-rate constant due to a substitution seems plausible if the exchanged residue poses a significant sterical hindrance for binding or if the complex formation proceeds via a two-step mechanism, e.g. if it involves a slower conformational transition from an initial low-affinity to a high-affinity complex. In the latter case, a mutation could interfere with the transition rate and thereby increase the time during which the complex remains in its low-affinity state. Both explanations, however, seem not to apply to the obscurin Ig58-Ig59/titin Z8-Z9 complex. While we have no kinetic data on the binding mechanism, R4444 is a very peripheral surface residue, and whose substitution by tryptophan exerts only very localized effects on chemical shifts (according to Rossi *et al*.’s NMR data (13)), suggesting that it does not alter the structural dynamics of obscurin Ig59. It is worth pointing out, however, that the SPR dissociation curves presented by Rossi *et al*. are not homogenous and therefore might have affected the quality of the fitting procedure. In agreement with Rossi *et al*., however, we did not find a biologically relevant effect of the R4444W variant on domain stability.

## Conclusion

Our results highlight the importance of interpreting *in vitro* data on potentially disease-causing variants in the context of human physiology and population genetics, and requiring experimental designs with sufficiently quantitative information. Our quantitative biochemical and biophysical characterization of the influence of the R4344Q or R4444W variants on protein–protein interactions and thermostability suggests that these variants are benign. Obviously, we cannot completely exclude the possibility that there are other protein–protein interactions or epistatic effects due to which the R4344Q or R4444W variants could exert a pathogenic influence, but these would not be covered by the mechanistic explanations of their actions on known interactions. For the R4344Q variant, however, this possibility seems unlikely in light of the overwhelming abundance amongst African Americans. Moreover, both variants were discovered in co-segregation with additional variants, suggesting that the disease phenotype observed in these patients could be the effect of multiple, individually benign, but in accumulation pathogenic variants. For the R4444W variant, this seems possible, having been discovered in patients with an FLNC frameshift mutation. For the R4344Q variant, our *in vitro* data suggest that even co-segregation with the A4484T variant may not lead to major increases in the risk for cardiovascular disease. While this does not rule out a minor risk increase; e.g. in the context of chronic heart disease due to lifestyle or environmental factors, we expect such predisposition not to be discernible without clinical studies of sufficient statistical power.

We, therefore, advise against classifying these variants as pathogenic and highlight the need to view genetic data in light of sufficient clinical and functional evidence that establishes a clear correlation between variants and the occurrence of cardiovascular or neuromuscular disease.

## Material and Methods

### Constructs used in this work

Obscurin Ig58 and Ig58-Ig59 (residue number 4337–4429 and 4337–4521, based on NCBI Ref. Seq. NM_001098623/NP_001092093, transcript variant B), titin Z8-Z9 and titin M2-M3 (1698 to 1931 and 34258 to 34454 based on NM_001267550/NP_001254479, transcript variant IC) and novex-3 obscurin binding region (ObB) (4991 to 5187 based on NM_133379/NP_596870, transcript variant novex-3) were cloned from a homemade human cardiac cDNA library into a modified pET protein expression vector encoding an N-terminal His_6_ tag followed by TEV cleavage site, and pEGFPC2 vector for expression in mammalian cells. The Arg4344Gln, Arg4444Trp and Ala4484Thr mutations were introduced by site-directed mutagenesis. pcDNA3-PLN was a kind gift of Dr. Joachim P. Schmitt (HHU Düsseldorf). All constructs were validated by sequencing.

### Protein expression and purification

Expression vectors were transformed into BL21-CodonPlus (DE3)-RIPL competent cells (Agilent Technologies), which were grown in 0.5 L of LB medium at 37^o^C until 0.6–0.7 at OD_600_ before protein expression was induced overnight by addition of 0.3 mM IPTG at 20°C. Cells were harvested by centrifugation and lyzed by sonication in PBS supplemented with lysozyme, 1:1000 (v/v) ß-mercaptoethanol, 30 mM imidazole, 1x cOmplete™ EDTA-free Protease Inhibitor Cocktail (Roche). Proteins were purified using 1ml superflow Ni-NTA resin (Qiagen) according to the manufacturer’s instructions and subsequently concentrated to 1.7–12 mg/ml (absorbance at 280 nm) into a final buffer of 30 mM HEPES pH 7.5, 100 mM NaCl and 2 mM DTT. Synthetic human full-length PLN, PLN_1-22_ (cytoplasmic region) and PLN_23-52_ (transmembrane region) was obtained from Pepscan (Lelystad, Netherlands) as lyophilized peptides and were resuspended in PBS supplemented with 0.2% (v/v) Triton X-100 and 2 mM DTT, or in 50 mM Tris–HCl pH 7.5, 100 mM NaCl, 0.5 % (v/v) Triton X-100 and 2 mM DTT. Due to interference of Triton X-100 with other methods, peptide concentration was determined densitometrically from SDS-PAGE using a BSA standard of known concentration and Oriole™ Fluorescent Gel Stain (Bio-Rad). All proteins were snap-frozen in liquid N_2_ and stored at −80^o^C until further use.

### Cell culture and transfection

HEK293A cells were cultured in DMEM supplemented with 10% foetal calf serum and 1% penicillin/streptomycin in 37°C/5% CO_2_ incubator. Around 70 % confluence, cells were transfected with plasmids using Escort IV (Sigma) following the manufacturer’s instruction. 24 h after transfection, cells were either fixed with 4% paraformaldehyde (PFA) for imaging, or lyzed with lysis buffer for pull-down assays.

### Immunostaining and imaging

Cells were fixed with 4% paraformaldehyde (PFA) in PBS for 6 min, permeabilized with 0.1% (v/v) Triton X-100 in PBS for 8 min, blocked with 5% normal goat serum/1% BSA in PBS for 30 min, and then treated with mouse anti-phospholamban antibody (primary, A1, Badrilla, 1:300 dilution) in 1% BSA in PBS for 2 h, followed by washing with PBS. Cells were then treated with Cy3-conjugated goat anti-mouse IgG (H+L) (Jackson ImmunoResearch, 115-165-146) in 1% BSA/PBS for overnight at 4^o^C. Following sufficient washing with PBS, the sample was mounted in mounting medium (30 mM Tris–HCl pH 9.5, 70% (v/v) glycerol, 5% n-propyl gallate). All procedures were performed at room temperature (RT). Cell images were collected on a Zeiss LSM510 confocal microscope in sequential scanning mode, using an APOCHROMAT 63x oil-immersion objective, instrument zoom rates set to 2 and image size of 1024 × 1024 pixels.

### Pull-down experiments

*Obscurin: phospholamban interaction.* About, 50 μl of superflow Ni-NTA beads (Qiagen) were equilibrated with 0.5 ml of assay buffer (40 mM imidazole in PBS, pH 7.4) three times. 100 μl of 8 nmol of His_6_-tagged proteins were mixed with the beads and incubated for 30 min at RT with moderate shaking to promote the binding. Unbound bait protein was removed by washing with 0.5 ml of assay buffer 3 times. 1 mg/ml PLN in 50 mM Tris–HCl pH 7.5, 100 mM NaCl, 2 mM DTT, 0.5 % (v/v) Triton X-100 was diluted with assay buffer to 0.2 mg/ml (32.8 μM), of which 100 μl were mixed with beads coated with bait protein, and then incubated for 1 h at RT with moderate shaking. A sample of the unbound fraction was taken (dilution of 1: 25 in 2x SDS sample buffer), and then beads were washed three times with 0.5 ml of washing buffer (PBS pH 7.2, 10 mM sodium azide, 0.1 % (v/v) Tween 20 as described in (14) supplemented with 40 mM of imidazole to reduce unspecific binding to the beads). The bound fraction samples were prepared by suspending the beads in 50 μl of 2x SDS sample buffer. 10 μl of bound and 3 μl of unbound fractions were loaded and separated by SDS–PAGE, transferred to a nitrocellulose membrane and analyzed by western blotting with mouse anti-PLN (A1, Badrilla, dilution of 1:5000) and HRP-conjugated rabbit anti-mouse IgG (Dako P0260, dilution of 1:1000). The signals of HRP were developed by Amersham^™^ ECL^™^ Western Blotting Detection Reagents (GERPN2109) and then imaged on a ChemiDocTM XRS+ imaging system (Bio-Rad).

*Obscurin: Novex-3 interaction.* ABout, 24 h after transfections with pEGFPC2 constructs, cells on 35-mm dishes were washed once with PBS and then lyzed with 150 μl of lysis buffer (50 mM Tris–HCl pH 7.5, 40 mM imidazole, 100 mM NaCl, 2 mM DTT, 10 % (v/v) glycerol, 0.5 % (v/v) Triton X-100, 1 mM MgCl_2_, 2x PhosStop phosphatase inhibitor cocktail (Roche), 0.5 μM Staurosporin, 1x cOmplete™ EDTA-free Protease Inhibitor Cocktail (Roche)). Soluble fractions were collected by centrifugation, 15 000 × g at 4^o^C for 20 min. 50 μl of His_6_-O5859-coated Ni-NTA beads prepared as described above were equilibrated with Novex-3 assay buffer (PBS, 40 mM imidazole, 2 mM MgCl_2_, 3.55 mM ß-mercaptoethanol, 0.2% (v/v) NP-40, 1x cOmplete™ EDTA-free Protease Inhibitor Cocktail (Roche) and 1x PhosStop phosphatase inhibitor cocktail (Roche)) before mixing with 60 μl of cell lysates. Following incubation at RT with moderate shaking for 1 h, a sample of the unbound fraction was taken (dilution of 1: 10 in 2x SDS sample buffer), and then beads were washed three times with 0.5 ml of Novex-3 assay buffer. The bound fraction samples were prepared by suspending the beads in 50 μl of 2x SDS sample buffer. All samples were boiled at 95^o^C for 5 min immediately before gel electrophoresis. About, 8 μl of bound and 4 μl of unbound fractions were loaded and separated by SDS–PAGE, transferred to a nitrocellulose membrane and then analyzed by western blotting with mouse anti-GFP (clones 7.1 and 13.1, Roche 11814460001, dilution of 1:2000), followed by HRP-conjugated rabbit anti-mouse IgG (Dako P0260, dilution of 1:2000). The signals of HRP were developed by Amersham^™^ ECL^™^ Western Blotting Detection Reagents (GERPN2109) and then imaged on a ChemiDocTM XRS+ imaging system (Bio-Rad).

### MST

Obscurin Ig58-Ig59, Ig58-Ig59_R4344Q,_ Ig58-Ig59_R4444W_ and Ig58-Ig59_R4344Q, A4484T_ was labelled for MST using the Monolith NT Protein Labeling kit RED-NHS (NanoTemper) according to the manufacturer’s instructions. The unlabelled binding partners were diluted serially by a factor of 0.5 in MST-Buffer (PBS, 0.2% (v/v) Triton x-100, 2 mM DTT for PLN and PBS, 2 mM DTT for Titin Z8-Z9 and ObB) and mixed with an appropriate amount of the labelled partner to give a signal of 300–700 fluorescence units (typically 50–100 nM). Thermophoresis was measured on a Monolith NT.115 instrument (NanoTemper) using 10-μl glass capillaries (Brand, cat.no. PIP3114). Data were transformed into fractional saturation and exported using the MO. Affinity Analysis software (NanoTemper) to be fitted to a one-site binding model (*Y* = $\frac{\big[X\big]}{K_d+\big[X\big]}$, where *Y* denotes fractional saturation and [*X*] the concentration of the free binding partner) using GraphPad Prism software version 8.3. Fitted dissociation constants ${K}_d$ are expressed as mean ± standard error.

### DSF

Purified obscurin Ig58, Ig58_R4344Q_, Ig58-Ig59, Ig58-Ig59_R4344Q_, Ig58-Ig59_R4444W_, Ig58-Ig59_A4484T_, and Ig58-Ig59_R4344Q, A4484T_ were mixed with SYPRO orange (ThermoFisher) to a final concentration of 20 μM and 20X, respectively. 20 μl of each protein-SYPRO orange mix was pipetted into 4–5 wells of a white 96-well PCR plate (ThermoFisher) covered with optical PCR seals (Bio-Rad) and DSF was run according to Niesen *et al.*(16); briefly, the plate was loaded into a Stratagene Mx3005p and heated from 25 to 96°C, 1^o^C per minute, and fluorescent emission of SYPRO orange was measured at each degree °C at 610 nm following excitation at 492 nm. The data relating to the unfolding of the protein were automatically identified using a Microsoft Excel spreadsheet from *Niesen et al*., with the melting temperature, *Tm*, calculated in GraphPad Prism (v8.3) by fitting the curve to the Boltzman Equation $Y= LL+\frac{UL- LL}{1+\exp \big(\frac{Tm-x}{a}\big)}$, where LL and UL refer to the lower and upper fluorescent intensities, *Tm* is the melting temperature, *Y* is the fluorescence at *x*, *x* is the temperature and *a* is the slope of the curve at *Tm*.

### Statistical analysis

Statistical significance between two experimental groups was determined in GraphPad Prism (v8.3) using the two-tailed unpaired *T*-test. Sample sizes are given in the respective figures.

For analysis of the population age structure of R4344Q carriers, data on single nucleotide variant 1-228503566-G-A (GRCh37) were retrieved from the gnomAD database v2.1.1 (accessed 27 March 2020). Absolute frequencies were transformed into relative frequencies and X/Y bar charts were plotted in GraphPad Prism (v8.3) by assigning X the arithmetic mean of the boundaries of the respective bin (the >80 years bin was assigned the value 90 years). For comparison of the age structure of all exomes, heterozygote and homozygote carriers, the data were fitted to Gaussian distributions and means were compared using the extra square-of-sums *F* test in GraphPad Prism. *P*-values <0.05 were considered significant.

## Supplementary Material

SUPPLEMENTAL_DATA_ddab010
