# Supplementary material for: When is an obscurin variant pathogenic? The impact of Arg4344Gln and Arg4444Trp variants on protein–protein interactions and protein stability
Source: Hum Mol Genet. 2021 Jan 12;30(12):1131–41. doi: 10.1093/hmg/ddab010 (PMC8188405; doi:10.1093/hmg/ddab010)
Supplement: SUPPLEMENTAL_DATA_ddab010 [file supplemental_data_ddab010.pdf]

SUPPLEMENTAL DATA

When is an obscurin variant pathogenic? The impact of Arg4344Gln and Arg4444Trp variants on protein-protein interactions and protein stability

Atsushi Fukuzawa[1,†], Daniel Koch[1,†,*], Sarah Grover[1],
Martin Rees[1], Mathias Gautel[1,*]

[1]Randall Centre for Cell & Molecular Biophysics, King's College London, United Kingdom

[†] authors contributed equally and are listed alphabetically.

* corresponding author: daniel.koch@kcl.ac.uk, Randall Centre for Cell & Molecular Biophysics, New Hunt's House, 18-20 Newcomen Street, London SE1 1UL. Phone: +44 2078486438.

* corresponding author: mathias.gautel@kcl.ac.uk, Randall Centre for Cell & Molecular Biophysics, New Hunt's House, 18-20 Newcomen Street, London SE1 1UL. Phone: +44 2078486709.

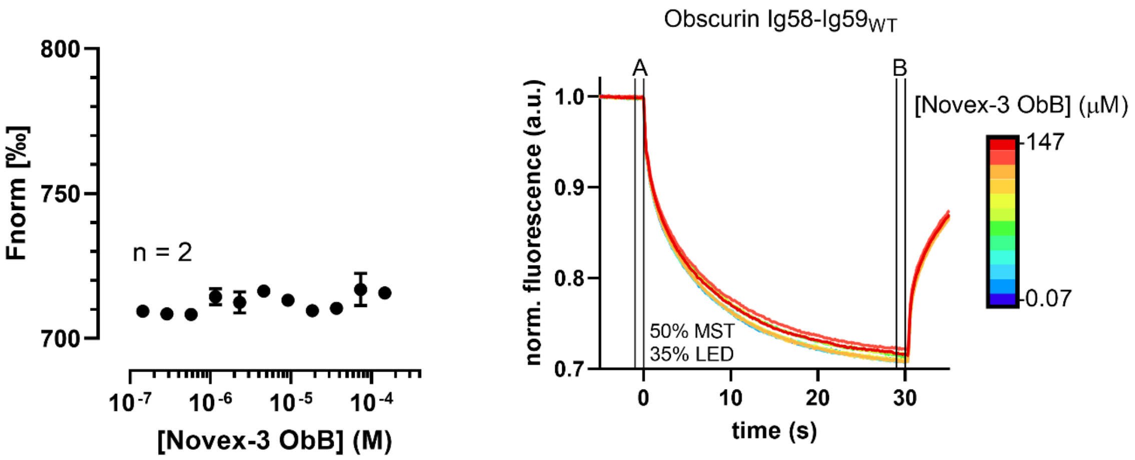

Figure S1: MST showed no binding between obscurin Ig58-Ig59 and recombinant novex-3 obscurin binding region (ObB). Dose response graph (left) based on signal at Lane B (MST-laser on) divided by signal at Lane A (MST-laser off) in the MST traces (right).

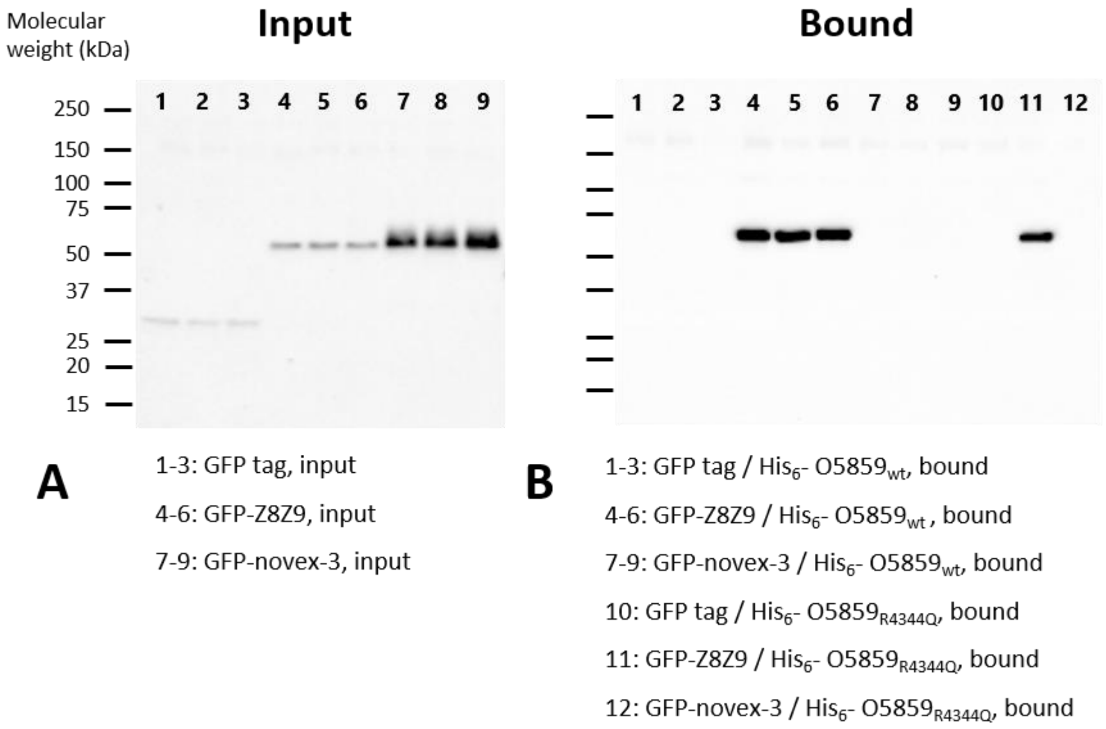

Figure S2: . Ni-NTA beads pull down assay. Titin novex-3 isoform showed no interaction with obscurin Ig58-Ig59. A) Cell lysates from HEK293A cells transfected with GFP-empty (lane1-3; negative control), GFP-Z8Z9 (lane4-6; positive control) or GFP-novex-3 ObB (lane7-9) were applied to Ni-NTA beads coated with His_6-O5859 wild type or R4344Q mutant. B) Bound fractions. Assays with His_6-O5859 wild type were triplicated.

```
CLUSTAL O(1.2.4) multiple sequence alignment


Bang et al. 2001    PPSSVEYFESPKSPDLYFNPSDITKQSSIHSGGETVERYSTPLGEVAERYSTPSEGEVGE 60
This study          -PSSVEYFESPKSPDLYFNPSDITKQSSIHSGGETVERYSTPLGEVAERYSTPSEGEVGE 59
                     ***********************************************************

Bang et al. 2001    RYSTPPGETLERYSTPPGETLERYSTPPGETLERYSTPPGETLERYSTPPGETLERYSTP 120
This study          RYSTPPGETLERYSTPPGETLERYSTPPGETLERYSTPPGETLERYSTPPGETLERYSTP 119
                    ************************************************************

Bang et al. 2001    PGEALERYSIPTGGPNPTGTFKTYPSKIEMEDGTPNEHFYTPTEERGSAYEIWRSDSFGT 180
This study          PGEALERYSIPTGGPNPTGTFKTYPSKIEREDGTPNEHFYTPTEERGSAYEIWRSDSFGT 179
                    ***************************** ******************************

Bang et al. 2001    PNEAIEPKDNEMPPSFIE   198
This study          PNEAIEPKDNEMPPSFIE   197
                    ******************
```

Figure S3: Alignment of the ObB sequence from novex-3 used in Bang *et al.* 2001 versus the sequence used in this study. While our sequence is shorter by one N-terminal proline (first arrow), the ObB sequence used in Bang et al. 2001 features a methionine at position 5139 instead of arginine. This residue is arginine in NM_133379 (transcript variant novex-3, titin) and methionine is reported as missense variant (dbSNP ID = rs66677602, AGG > ATG, minor allele frequency = 0.0773, ClinVar evaluation = benign).

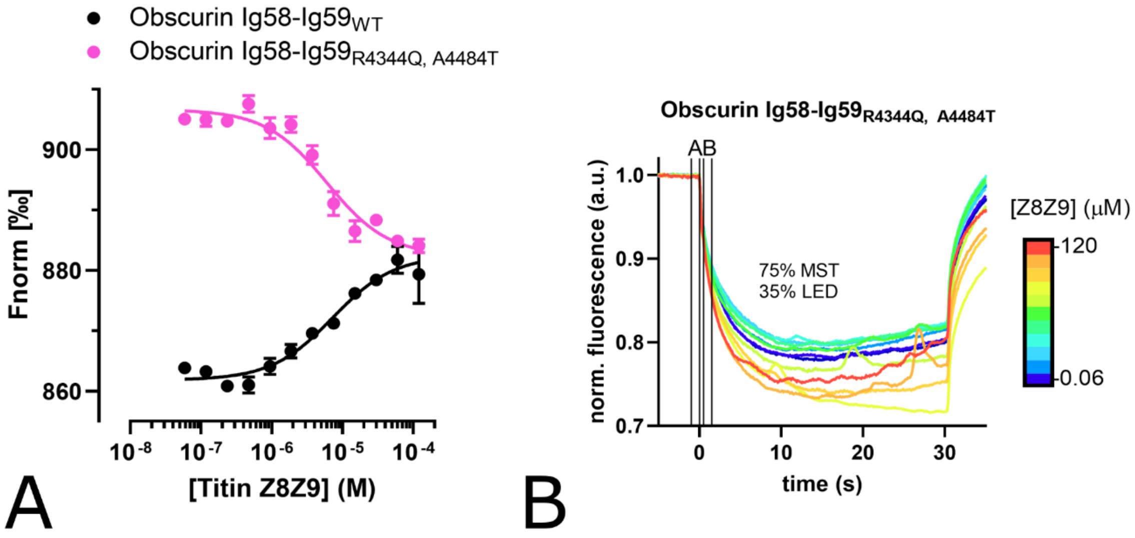

Figure S4: (A) The A4484T variant (pink) causes a decrease in the normalised fluorescence of the temperature jump signal upon ligand binding, whereas WT (black) and R4344Q and R4444W variants (not shown) lead to increased fluorescence upon ligand binding. (B) Bumpy segments in the later parts of the MST traces indicate formation of aggregates

Supplementary references:

Bang, M-L., Centner, T., Fornoff, F., Geach, A. J., Gotthardt, M., McNabb, M., Witt, C. C., Labeit, D., Gregorio, C. C., Granzier, H., et al. (2001) The Complete Gene Sequence of Titin, Expression of an Unusual ≈700-kDa Titin Isoform, and Its Interaction With Obscurin Identify a Novel Z-Line to I-Band Linking System. *Circ. Res.*, **89**, 1065–1072.
